# *NUS1* and Epilepsy-myoclonus-ataxia Syndrome: An Under-recognized Entity?

**DOI:** 10.5334/tohm.696

**Published:** 2022-06-15

**Authors:** Giulietta M. Riboldi, Edoardo Monfrini, Christine Stahl, Steven J. Frucht

**Affiliations:** 1 The Marlene and Paolo Fresco Institute for Parkinson’s and Movement Disorders, Department of Neurology, NYU Langone Health, New York, NY, USA; 2 Dino Ferrari Center, Neuroscience Section, Department of Pathophysiology and Transplantation, University of Milan, Milan, Italy; 3 IRCCS Ca’ Granda Ospedale Maggiore Policlinico, Neurology Unit, Milan, Italy

**Keywords:** myoclonus, ataxia, NUS1, myoclonus epilepsy, myoclonus-ataxia, genetics

## Abstract

**Background:**

Variants of the *NUS1* gene have recently been linked to a spectrum of phenotypes including epilepsy, cerebellar ataxia, cortical myoclonus and intellectual disability (ID), and primary congenital defects of glycosylation.

**Case Report:**

We report a case of myoclonus epilepsy, mild cerebellar ataxia, and ID due to a new de-novo *NUS1* missense variant (c.868C>T, p.R290C), and review the current literature of *NUS1*-associated clinical phenotypes.

**Discussion:**

Pathogenic variants of *NUS1* are found in a rapidly growing number of cases diagnosed with myoclonus epilepsy and/or myoclonus-ataxia syndrome. *NUS1* should be included in the genetic screening of undiagnosed forms of myoclonus, myoclonus-ataxia, and progressive myoclonus epilepsies.

## Introduction

The *NUS1* gene encodes for the Nogo-B receptor (NgBR) which stabilizes the Dehydrodolichyl-diphosphate synthase complex in the endoplasmic reticulum, promoting its enzymatic activity (cis-PTase). Recessive pathogenic variants of this gene were first described in two siblings presenting with a congenital defect of glycosylation (CDG) [1]. CDG are conditions characterized by psychomotor retardation, cerebellar hypoplasia, facial and ocular dysmorphism, retinitis pigmentosa, and skin lesions [2]. After this initial description, a growing number of cases carrying fully penetrant autosomal dominant *NUS1* variants have been reported worldwide in patients presenting with a spectrum of phenotypes including epilepsy, cerebellar ataxia, cortical myoclonus, intellectual disability, and psychomotor developmental delay [3,4,5,6,7,8,9,10,11,12].

Here we report a new case presenting with epilepsy, myoclonus, mild cerebellar ataxia and intellectual disability carrying a novel heterozygous *NUS1* missense variant.

## Case report

We evaluated a 28-year-old African-American woman with a history of myoclonus and epilepsy. She was born at term from an uncomplicated vaginal delivery, with initial normal development. A teacher noticed “hand tremors” at the age of 4 years. She had normal socialization and a mild learning disability but was able to finish high school and start college. At the age of 13, she developed her first generalized myoclonic seizure, followed by multiple episodes of early morning focal onset seizure with secondary generalization and additional episodes of generalized myoclonic seizures. She was treated with zonisamide and then topiramate with good control of the seizures. A previous EEG showed diffuse excessive fast activity. Brain MRI showed mild cerebellar atrophy (Figure 2).

The “hand tremor” was later identified as myoclonus and worsened during her high school years, with prominent involvement of her upper limbs. She was treated with clonazepam with moderate benefit. At her last evaluation, she displayed multifocal myoclonus at rest, mostly involving her face and distal upper limbs, mild action myoclonus at target, and no clear stimulus sensitivity. There were only very mild cerebellar signs (including mild saccadic pursuits, dysdiadochokinesia, and appendicular dysmetria) with no significant gait impairment (Video 1). Patient’s cognitive profile was not formally assessed but she presented a decline over the years affecting her school career. Her parents were both from Antigua. There was no consanguinity in the family and no family history of seizures or other neurological conditions. Written authorization for the acquisition of the video for publication for scientific purposes was signed by the patient.

**Video 1:** **Case presentation: clinical features of *NUS1* and MEAD syndrome.** The video shows the most relevant clinical features of a new case of myoclonus-epilepsy associated with a novel de novo missense variant of *NUS1* (c.868C>T, p.R290C): speech is preserved; there is multifocal, mini-myoclonus of the face; eye movements only showed mild saccadic intrusion of pursuits; there is appendicular myoclonus involving the upper limbs, distally; there is no bradykinesia and only mild incoordination; there is action myoclonus that increases at target in both upper limbs; there is only mild appendicular ataxia and past-pointing; there is no stimulus-sensitive myoclonus; there is significant action myoclonus at approaching the paper with a pen and drawing a spiral; gait is narrow-based with no ataxia.

Previous genetic testing, including 21 genes of progressive myoclonic epilepsy and Dentatorubro-Pallidoluysian Atrophy (DRPLA) expansion, were negative. Targeted gene testing of *NUS1* revealed a novel missense mutation of the *NUS1* gene, c.868C>T (p.Arg290Cys), initially classified as a variant of unknown significance (VUS). The variant was not found in large population datasets [13] and was absent in her parents (*de novo*). Therefore, the p.Arg290Cys was re-classified as likely pathogenic (ACMG criteria: PM1, PM2, PM5, PM6, PP3). This variant affects the same residues reported in previous cases of CDG with recessive mode of inheritance (p.R290H). We hypothesize that the different aminoacidic change in our case (p.R290C) may have a more profound impact on an important domain of the NUS1 protein due to the biochemical differences between Arginine and Cysteine, compared to Arginine and Histidine.

To the best of authors’ knowledge and as per Genetic Testing Registry (GTR, https://www.ncbi.nlm.nih.gov/gtr/) there are no gene panels for the myoclonic epilepsy in the US that include *NUS1*.

## Review of the literature

**Figure 1:**
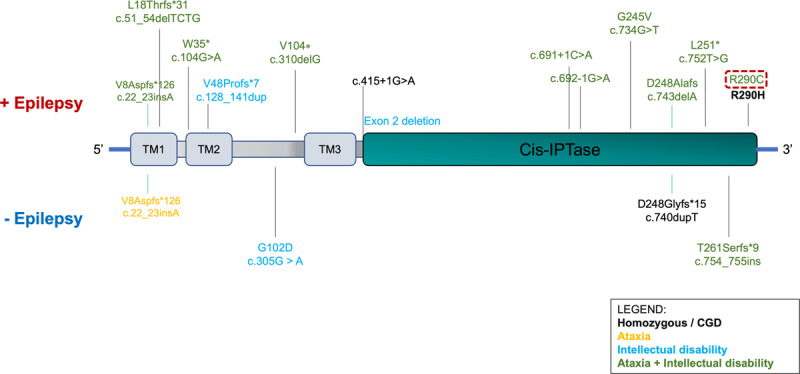
***NUS1* pathogenic variants and related phenotypes.** The figure summarizes the pathogenic variants reported in the literature in the NUS1 gene, highlighting their position on the gene, and associated phenotype: ataxia (orange), ID (light blue), or both (green) with epilepsy (upper part of the figure) or without epilepsy (lower part of the figure). Myoclonus was reported in all the listed variants except for c.869G>A (p.Arg290His) (homozygous), and c.743delA (p.Asp248Alafs). The only reported homozygous variant associated with CDG is bolded. Protein domains are labeled. TM: transmembrane. The new variant found in this report (likely pathogenic) is highlighted by the red box.

So far, 22 cases of patients with *NUS1* variants with variable clinical presentations have been reported in the literature. Here we summarize the most significant phenotypes (Figure 1). A detailed summary table including clinical and demographic features of patients with *NUS1* variants was recently reported [12].

**Figure 2:**
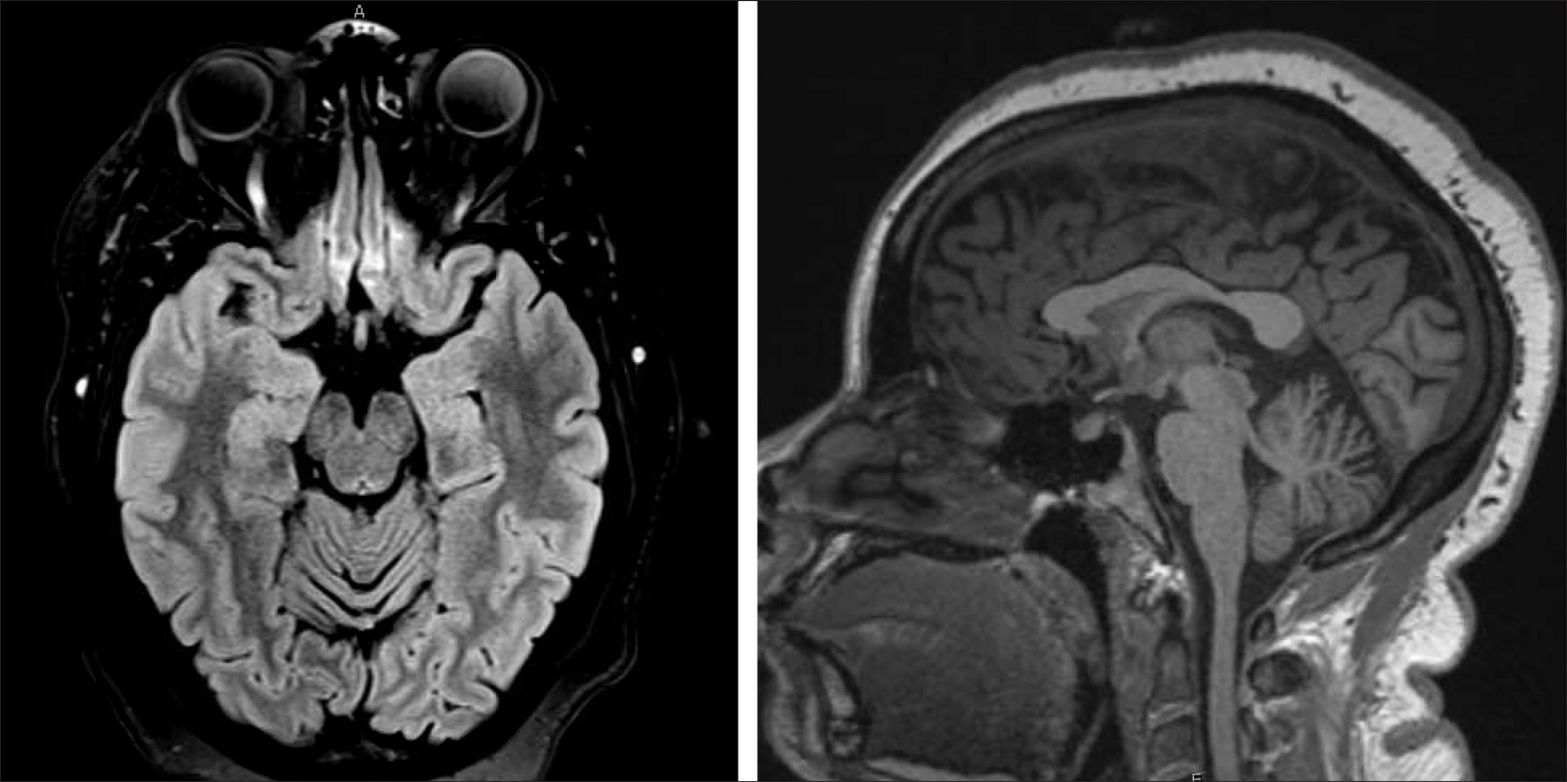
**Brain MRI of the described case.** The images show axial FLAIR (on the right) and coronal T1 (on the left) brain MRI imaging of this case, at age 25. Both images showed a mild cerebellar atrophy.

### NUS1 and defects of glycosylation

Homozygous variants of *NUS1* have been reported in only one family with CDG type 1 [1]. This was a Roma family where two siblings out of four presented with a complex syndrome characterized by psychomotor delay, delayed uterine growth, hypotonia at birth, congenital scoliosis, hearing and vision impairment as well as macular lesions. Between the 7^th^ and 11^th^ month of life these subjects also developed refractory epilepsy with generalized tonic-clonic seizures. One of the two brothers died at the age of 19 months, and the other survived after the age of 4 years with pseudobulbar palsy, appendicular spasticity, microcephaly, failure to thrive, and hypertrichosis. Brain MRI in this proband showed cortical atrophy. Whole exome sequencing (WES) in the probands identified a homozygous missense *NUS1* variant (c.869G>A, p.Arg290His). Functional analysis in patient-derived fibroblasts showed a loss of function of the variant, causing accumulation of free cholesterol - similarly to conditions where NgBR is silenced [14] -, reduced cis-PTase activity and mannose incorporation into proteins, as well as hypo-glycosylation of target proteins, such as LAMP-1 and ICAM-1. Additional pathogenic variants of *NUS1* associated with CDG, as reported in ClinVar, are summarized Table 1.

**Table 1:** **Additional pathogenic *NUS1* variants associated with CDG reported in ClinVar.** Detailed phenotype description was not available for these variants.


VARIANT	PROTEIN CHANGE	CONDITION(S)	CLINICAL SIGNIFICANCE	SOURCE

NM_138459.5(NUS1):c.15C>A (p.Tyr5Ter)	Y5*	Congenital disorder of glycosylation, type IAA	Pathogenic	ClinVar

NM_138459.5(NUS1):c.74_75delinsAA (p.Trp25Ter)	W25*	Congenital disorder of glycosylation, type IAA	Pathogenic	ClinVar

NM_138459.5(NUS1):c.74G>A (p.Trp25Ter)	W25*	Congenital disorder of glycosylation, type IAA	Pathogenic	ClinVar

NM_138459.5(NUS1):c.99dup (p.Asn34fs)	N34fs	Congenital disorder of glycosylation, type IAA	Pathogenic	ClinVar

NM_138459.5(NUS1):c.238_263del (p.Ala80fs)	A80fs	Intellectual disability, autosomal dominant 55, with seizures	Pathogenic	ClinVar

NM_138459.5(NUS1):c.415+1G>A		Intellectual disability, autosomal dominant 55, with seizures	Pathogenic	ClinVar

NM_138459.5(NUS1):c.443T>A (p.Leu148Ter)	L148*	Inborn genetic diseases	Pathogenic	ClinVar

NM_138459.5(NUS1):c.719T>G (p.Leu240Ter)	L240*	Congenital disorder of glycosylation, type IAA	Pathogenic	ClinVar


### NUS1 and myoclonus, epilepsy, ataxia, intellectual disability syndrome (MEAID syndrome)

In a larger cohort of subjects with heterozygous pathogenic variants of *NUS1*, frequently with de-novo occurrence, the phenotype was characterized by various combinations of epilepsy, intellectual disability, cerebellar ataxia, and cortical myoclonus (MEAID). Reported subjects were European, French-Canadian, Japanese, Chinese, and African-American [3,4,5,6,7,8,9,10,11,12]. Pathogenic variants included missense, frameshift, and truncating variants.

Looking at this cohort, common clinical features help define a phenotype associated with *NUS1* variants. Disease onset was early in life (from a few months to 13 years of age). These patients frequently presented with cortical myoclonus which was multifocal, mostly appendicular, with a component at rest and with action. When present, myoclonus of the limbs preceded the onset of seizures. Interestingly, a multifocal, mini-myoclonus of the face has been observed in different probands (including our case, [11,12]) and can be an important clue for suspecting variants of this gene. Stimulus-sensitivity was not assessed in the majority of the cases. In two reports, myoclonus seemed to respond well to baclofen [4,11]. Epilepsy was usually characterized by a combination of generalized tonic-clonic, absence, and myoclonic seizures. Seizure control may require polypharmacy but there are no reports of treatment-refractory epilepsy. Cerebellar ataxia was usually mild and mainly appendicular, with less involvement of gait, often associated with scanning speech. Interestingly, cases of myoclonus without overt ataxia have also been reported [10,12]. Intellectual disability was noticed early in life, often mildly progressive but usually not incapacitating. However, in a few patients, severe intellectual disability was described [3]. Of note, the genetic variants in these two subjects were predicted to affect the C-terminal domain of *NUS1* which is responsible for interacting with the Dehydrodolichyl Diphosphate Synthase Subunit (DHDDS) and thus severely affects its function [2]. Other rarer features, such as dystonia, psychotic symptoms, parkinsonism, and scoliosis have been reported [4,5,6,7].

Follow-up studies suggest a slowly progressive worsening of the cognitive features [8]. The longest follow-up (up to the age of 59 years) has been reported by Den et al. [4]. The subject they described presented with an early-onset phenotype characterized by myoclonus, seizures, and mild intellectual disability. However, later in life, cerebellar and cognitive symptoms became severe, and myoclonus seemed to be less responsive to medications.

Imaging studies in these patients, including brain MRI, were usually normal or showed mild cerebellar atrophy. Thickening of the corpus callosum was reported in only one case [6].

### NUS1 and PD

Enrichment of rare *NUS1* variants in patients with Parkinson’s disease (PD) was described in a WES study of subjects with early-onset PD and in a second large study assessing *NUS1* variants in 1542 PD cases vs 1625 controls [15,16]. However, follow-up studies, including burden analysis of rare nonsynonymous damaging variants of *NUS1* in WES and WGS datasets, analysis of large PD-GWAS for rare and common variants of *NUS1*, and full *NUS1* sequencing in a large cohort of PD subjects failed to validate enrichment of *NUS1* variants in subjects with PD [17,18,19].

## Conclusion

Since the initial identification of recessive *NUS1* gene variants in subjects with CDG, a growing number of patients harboring heterozygous variants of this gene have been reported. Autosomal dominant, mostly *de novo* variants of this gene have been associated with a constellation of symptoms that we term MEAID [3,4,5,6,7,8,9,10,11,12]. Epilepsy is usually well managed with anti-epileptic medications. Large long-term follow-ups are still lacking for appropriate counseling of these patients. Here we report an additional case of myoclonus, epilepsy and mild intellectual disability and ataxia associated with a novel likely pathogenic *NUS1* variant. This proband remained undiagnosed for many years as the *NUS1* gene is not included in the majority of myoclonus epilepsies panels, which was the prominent phenotype of this subject.

In conclusion, *NUS1*-associated disease may be an under-recognized entity and we suggest that the *NUS1* gene should be included in the genetic screening for myoclonus epilepsy as well as MEAID syndrome.
